# Improved Sleep Quality and Sleep Duration After an 8-Week Exergaming Intervention for Exercise Training Among Elementary Schoolchildren in Taiwan

**DOI:** 10.3390/children12020180

**Published:** 2025-01-31

**Authors:** Hsiao-Fang Kao, Chi-Fang Lin, I-Tung Lin, Yi-Chuan Hung, Ting-Wei Chang, Chien-Chang Ho

**Affiliations:** 1 Department of Sports Management, Minghsin University of Science and Technology, Hsinchu 304001, Taiwan; joyce@must.edu.tw; 2 Department of Physical Education and Sport Sciences, National Taiwan Normal University, Taipei 106308, Taiwan; aa830521@gmail.com; 3 Office of Physical Education, Fu Jen Catholic University, New Taipei 24205, Taiwan; yichuan1206@gmail.com; 4 College of General Education, Chihlee University of Technology, New Taipei 22050, Taiwan; dachandie@mail.chihlee.edu.tw; 5 Department of Sport Management, National Taiwan University of Sport, Taichung 404401, Taiwan; 6 Sports Administration, Ministry of Education, Taipei City 104703, Taiwan; 7 Department of Physical Education, Fu Jen Catholic University, New Taipei 24205, Taiwan; ctw150160@gmail.com; 8 Sports Medicine Center, Fu Jen Catholic Hospital, New Taipei 24352, Taiwan

**Keywords:** active video sports game, sleep quality, sleep duration, elementary school student

## Abstract

Objectives: The present study aimed to investigate the effects of an 8-week exergaming intervention for exercise training on sleep quality and sleep duration among elementary schoolchildren in Taiwan. Methods: A randomized controlled trial design was employed, with 68 elementary schoolchildren (aged 10.76 ± 0.49 years) recruited and randomly assigned to either an experimental group (*n* = 35) or a control group (*n* = 33). The experimental group participated in an 8-week exergaming intervention for exercise training; this comprised three sessions per week consisting of three rounds per session, with each round lasting 1 min. The control group did not receive any training and maintained their regular daily routines. All participants provided information on their demographic characteristics and anthropometric measurements and completed the Pittsburgh Sleep Quality Index (PSQI) questionnaire at both pre-test (baseline) and post-test (week 8) assessments. Results: After 8 weeks of exergaming intervention, the experimental group showed a significant reduction in the PSQI global score (6.17 ± 2.96 vs. 4.80 ± 1.97, *p* < 0.0001). The change in the PSQI global score in the experimental group was significantly greater than in the control group (−1.37 ± 1.97 vs. −0.27 ± 1.89, *p* = 0.022). Additionally, the experimental group demonstrated a significantly longer sleep duration at week 8 compared with the control group (505.15 ± 53.16 vs. 480.51 ± 54.13, *p* = 0.047). Conclusions: Overall, the findings of this study indicate a beneficial response of elementary schoolchildren to exergaming, with participants reporting improved sleep quality and sleep duration as a result of engaging in the exergaming intervention sessions three times per week for 8 weeks, with three one-minute rounds per session. Therefore, it appears that exergaming may be an effective method for improving certain aspects of sleep quality and sleep duration among children between 10 and 12 years of age.

## 1. Introduction

In recent years, sleep quality issues among children and adolescents have become increasingly prominent. Previous research has indicated that the prevalence of poor sleep quality ranges from 11% to 47% [1,2]. Additionally, studies have suggested that children and adolescents require an average of 9 h of sleep per night, yet 45% of them sleep for less than 8 h [3,4]. Sleep is closely related to both physical and mental health, playing a crucial role in the growth, cognitive development, learning performance, and neurobehavioral functions of children and adolescents [5,6,7,8]. Research has shown that insufficient or disrupted sleep may reduce nocturnal brain activity, consequently impairing cognitive functions, particularly abstract thinking, creativity, integration, and planning [9]. Therefore, poor sleep quality or insufficient sleep during adolescence may compromise the executive functions of the prefrontal cortex [5,10,11], leading to declines in learning ability and academic performance [12,13].

While sleep quality and sleep duration are somewhat related, they are qualitatively distinct and, thus, are treated as separate domains. Sleep quality refers to an individual’s subjective experience of sleep, including the feeling of restfulness upon waking and satisfaction with sleep. Sleep duration, on the other hand, refers to the total amount of time spent sleeping [4]. The correlation between sleep quality and sleep duration in children and adolescents is relatively low, supporting the notion that they represent two independent sleep domains [14,15]. Although previous studies have found both domains to be related to factors such as fatigue, mood, behavior, and cognitive functioning [8,11], sleep quality has been found to have a stronger association with these factors than sleep duration [4].

The relationship between physical activity and sleep has been a subject of significant interest. A meta-analysis revealed that both short-term and long-term exercise interventions are associated with improved sleep, which is characterized by shorter sleep onset latency and enhanced sleep efficiency [16]. Additionally, research on adolescents has shown that athletes typically exhibit better sleep quality compared with non-athletes [17]. Further studies found that adolescents with longer weekly exercise durations tend to have better sleep quality than those with shorter exercise durations [18]. In a randomized trial, 27 adolescents who participated in 30 min of morning running on school days showed significant improvements in both objective and subjective sleep quality compared with 24 non-participating peers [19]. While the relationship between physical activity and sleep shows a promising trend, further research is required for a deeper understanding.

Since 2022, Taiwan’s Ministry of Education has promoted the “Digital Learning Enhancement Program for Primary and Secondary Schools”, which expands digital learning policies across schools. In this context, exergaming has garnered increasing attention and has become an important educational tool. One study involving the Wii Fit found that children’s oxygen uptake significantly improved after three months of gaming [20]. Moreover, Staiano et al. [21] conducted a 24-week exergaming intervention with overweight and obese children, having them engage in three 1-hour sessions per week. The results showed that exergaming effectively improved BMI, cardiometabolic health, and physical activity levels. Similarly, a retrospective study by Comeras-Chueca et al. [22] systematically reviewed the effects of active video games (AVGs) and found that interventions lasting over 18 weeks resulted in improvements in children and adolescents’ BMI and chronic kidney disease, suggesting that the use of AVGs could be a health-improving strategy. Lastly, a narrative review by Calcaterra et al. [23] highlighted the potential of exergaming to influence body composition, weight management, and sedentary behavior changes. Given that exergaming appears to have outcomes similar to traditional exercise, this study examines the effects of an 8-week exergaming intervention for exercise training on sleep quality and sleep duration among elementary schoolchildren in Taiwan.

## 2. Materials and Methods

### 2.1. Study Design and Ethics Approval

This study was designed as a randomized controlled trial with an exergaming intervention period of 8 weeks. Demographic characteristics (grade, age, and gender), anthropometric measurements (height, body weight, and body mass index [BMI]), and the Pittsburgh Sleep Quality Index (PSQI) questionnaire were assessed prior to (pre-test) and following (post-test) the 8-week period of exergaming intervention or control conditions. Before the commencement of this study, the exercise protocol, testing procedures, and benefits and possible risks were explained in full to ensure that all participants and their parents or legal guardians understood the research process. An informed consent form was signed by the parents or guardians. This study conformed to the Declaration of Helsinki and was approved by the Institutional Review Board of Fu Jen Catholic University in Taiwan (FJU-IRB C112169).

### 2.2. Participants

Sixty-eight elementary schoolchildren aged 10–12 years old were included in this study. The inclusion criteria for voluntary participation were as follows: (1) fifth- and sixth-grade male and female schoolchildren (33 boys and 35 girls) and (2) non-regular exercise behavior. Participants were excluded if they met any of the following conditions: (1) any disease or clinical symptoms requiring medical attention; (2) any neurological, muscular, or skeletal system injuries within the past 6 months; and (3) an affirmative answer to any question on the Physical Activity Readiness Questionnaire (PAR-Q). Participants were randomly assigned to either the experimental group (*n* = 35) or the control group (*n* = 33). During the exergaming intervention period, the researchers contacted the participants weekly via phone to inquire about their physical activity and dietary intake. The participants were encouraged to maintain their usual exercise routines and dietary habits to minimize inter-individual variation in daily physical activity and food intake.

### 2.3. Data Collection

A self-administered questionnaire survey and anthropometric measures were completed by well-trained research assistants. Demographic data, including age, gender, and grade, were recorded. Anthropometric measures were carried out after the participants had removed their shoes and heavy clothes. Body weight was measured to the nearest 0.1 kg, and body height to the nearest 0.1 cm. The body mass index (BMI) was calculated as weight in kilograms divided by height in meters squared. Children’s BMIs were categorized based on age and sex-specific cut-off values from the Health Promotion Administration, Ministry of Health and Welfare, in Taiwan [24]. The BMI categories were underweight (<5th percentile), normal weight (5th to 85th percentile), overweight (85th to 95th percentile), and obesity (>95th percentile).

### 2.4. Exergaming Intervention

The participants in the experimental group engaged in an 8-week exergaming intervention for exercise training. Sessions were conducted three times per week, with each session consisting of three rounds, each lasting 1 min. The exergaming intervention included three different games, with each round structured as follows: (1) Laser Dodge, where participants avoided red laser beams on the screen by moving their bodies. As the level of difficulty increased, additional lasers appeared, requiring continuous dodging movements, such as jumping and squatting; (2) Cone Knockout, where participants moved using lateral steps to knock over virtual cones on the left and right sides of the screen; (3) Box Attack, where participants had to position their bodies inside randomly appearing boxes on the screen to trigger the appearance of the next box. During the exergaming intervention for exercise training, the exercise intensities were performed at moderate- to vigorous-intensity levels (heart rate ≥ 130 beats/min). This game involves movements such as jumping, squatting, and adopting prone positions. Participants in the control group maintained their regular daily routines and refrained from engaging in any structured exercise throughout the intervention period.

### 2.5. Sleep Quality and Sleep Duration Assessment

This study utilized the Chinese version of the Pittsburgh Sleep Quality Index (PSQI) as a research tool for assessing sleep quality and sleep duration in children; the index was developed by Buysse [25] and translated by Tsai et al. [26]. Internal consistency and test–retest reliability of the PSQI have been tested and verified on a sample of children [27]. Cronbach’s alpha coefficient was 0.719, and the intraclass correlation coefficient was 0.829 (95% confidence interval: 0.662–0.914). The PSQI evaluates participants’ sleep quality over the past month using seven components: subjective sleep quality, sleep latency, sleep duration, sleep disturbances, sleep efficiency, use of sleeping medication, and daytime dysfunction. Each component is scored from 0 to 3, with a total score ranging from 0 to 21. A PSQI total score of over or equal to 5 has been validated as highly sensitive and specific for distinguishing poor sleep quality from good sleep quality. The PSQI was completed by the parents or guardians, with assistance from the children as necessary. Additionally, sleep duration was assessed using the following PSQI component: “During the past month, how many hours of actual sleep did you get at night?”. Responses on sleep duration by the parents or guardians were estimated in minutes based on how many hours they reported their participating children being actually asleep at night over the past month.

### 2.6. Statistical Analysis

Data processing and analysis were conducted using IBM SPSS Statistics 25.0 (SPSS Inc., Chicago, IL, USA). The mean, standard deviation (SD), and percentage were used as descriptive statistics for the participants’ characteristics and study outcome measurements. The demographic characteristics of the two groups were compared using the Student’s *t*-test for continuous variables and the chi-square test or Fisher’s exact test for categorical variables. Comparisons of sleep quality and sleep duration measures before and after the exergaming intervention within each group were performed using the paired *t*-test. Pre- and post-test differences and changes in sleep quality and sleep duration measures between the experimental group and the control group were assessed using the Student’s *t*-test. In addition, mixed-model analysis of variance (ANOVA) was carried out to test the effects of group, time, and interaction (group × time) for sleep quality and sleep duration measures. A *p* value < 0.05 was considered statistically significant.

## 3. Results

### 3.1. Participant Demographics

The statistical analysis results for the participants’ demographic characteristics and anthropometric measurements are presented in Table 1. The Student’s *t*-test results showed non-significant differences between the experimental and control groups for the continuous variables of age, height, body weight, and BMI (*p* > 0.05). The chi-square test results indicated a non-significant difference in the categorical variable of gender and sleep quality between the two groups (*p* > 0.05). Additionally, the Fisher’s exact test results indicated significant differences in the categorical variable of BMI between the two groups (*p* = 0.024). However, the experimental group had significantly higher percentages of overweight (8.82% vs. 3.13%) and underweight (73.54% vs. 12.50%) compared with the control group. On the other hand, the experimental group had significantly lower percentages of obesity (8.82% vs. 37.50%) and normal weight (8.82% vs. 46.87%) compared with the control group.

### 3.2. Sleep Quality

Table 2 presents the statistical analysis results comparing the pre-test, post-test, and change in sleep quality measures between the experimental group and the control group after 8 weeks of exergaming intervention. According to the Student’s *t*-test, there were non-significant differences between the two groups in the pre-test values for the PSQI global score (*p* > 0.05). In the post-test values, there were also non-significant differences between the two groups in the PSQI global score (*p* > 0.05). Regarding the change in PSQI global score between the pre-test and post-test assessments, the experimental group showed a significantly greater reduction in the PSQI global score compared with the control group (−1.37 ± 1.97 vs. −0.27 ± 1.89, *p* = 0.022), suggesting that the 8-week exergaming intervention contributed to an overall improvement in sleep quality as measured using the PSQI global score.

The paired *t*-test results showed that in the experimental group, there were significantly higher PSQI global scores (6.17 ± 2.96 vs. 4.80 ± 1.97, *p* < 0.0001) from the pre-test assessment to the post-test assessment, indicating that the 8-week exergaming intervention was effective in improving sleep quality.

Repeated measures ANOVA of PSQI global scores of the two groups before and after 8 weeks of exergaming intervention revealed statistically significant time (*p* = 0.001) and interactive (*p* = 0.0018) effects for the PSQI global score.

### 3.3. Sleep Duration

Table 3 presents the statistical analysis results comparing the pre-test and post-test assessments and changes in sleep duration measures between the experimental and control groups after 8 weeks of exergaming intervention. According to the Student’s *t*-test, there was a non-significant difference in sleep duration between the two groups at the pre-test assessment (*p* > 0.05). However, during the post-test assessment, the experimental group showed a significantly longer sleep duration than the control group (505.15 ± 53.16 min vs. 480.51 ± 54.13 min, *p* = 0.047), indicating that the 8-week exergaming intervention contributed to an increase in sleep duration. Regarding the change in scores between the pre-test and post-test assessments, there was a non-significant difference in sleep duration between the two groups (*p* > 0.05).

The paired *t*-test results showed a non-significant difference in sleep duration between the pre-test and post-test assessments within both the experimental and control groups (*p* > 0.05). Repeated measures ANOVA of sleep duration of the two groups before and after 8 weeks of exergaming intervention revealed non-statistically significant time and interactive effects for sleep duration (*p* > 0.05).

## 4. Discussion

This study is the first to observe the beneficial effects of an exergaming intervention for exercise training on sleep quality and sleep duration among elementary schoolchildren in Taiwan. The key findings from this 8-week exergaming intervention are as follows: after 8 weeks, the children’s sleep quality (measured using the total PSQI global score, sleep latency, sleep disturbances, and daytime dysfunction) and sleep duration showed significant improvements. Previous studies on exergaming also demonstrated similar outcomes. One study using Xbox 360 Kinect games for a 6-week intervention found potential improvements in sleep and mental health [28]. Another study reported significant improvements in total sleep time, the respiratory disturbance index, the apnea/hypopnea index, and the number of apnea episodes after 12 weeks of using Wii-based aerobic exercises [29]. Furthermore, neuroscience research indicates that regular physical activity can, on the one hand, enhance slow-wave activity in the brain during sleep and, on the other hand, decrease rapid eye movement (REM) sleep [30]. This improvement in sleep patterns contributes to better sleep quality and alleviates sleep disturbances, in addition to helping reduce tension and mental stress while stimulating the secretion of hormones in the brain.

This study may have benefited from the use of active video games, which not only combine elements of exercise and entertainment but also leverage advancements in modern technology to integrate physical activity into children’s daily recreational activities. Previous research has shown that although children have some awareness of the benefits of exercise, their primary motivation for participating in physical activities is often “fun” [31]. Conversely, a systematic literature review found that active video games can significantly increase children’s heart rates and energy expenditure. However, the long-term effects of these activities, categorized as light-to-moderate physical activity, remain inconsistent [32]. One study confirmed the exercise efficacy of a 24-week intervention with active video games, indicating small but significant impacts on BMI and body composition in overweight and obese children [33]. This suggests that active video games may be a promising method of promoting physical activity among children, although further research is required to explore their long-term effects.

Despite the positive association in this study between exergaming intervention and sleep quality, there are certain limitations. First, only elementary schoolchildren in Taiwan were observed, using a relatively small sample size limited to a specific age group and region, which may not represent children from other demographics or areas. Second, this study was designed as a randomized controlled trial without blinding, which may have introduced social desirability bias. Moreover, as the participants were volunteers, there was potential for self-selection bias and sampling bias, which could affect the reliability of the results. Therefore, future research could utilize more diverse recruitment methods to minimize the likelihood of sampling bias. Lastly, our study relied on self-report measures, which are subject to recall bias.

## 5. Conclusions

Overall, the findings of this study indicate a beneficial response of elementary schoolchildren to exergaming, with participants reporting improved sleep quality and sleep duration as a result of engaging in exergaming intervention sessions three times per week for 8 weeks, with three one-minute rounds per session. Therefore, it appears that exergaming may be an effective method for improving certain aspects of sleep quality and sleep duration among children between 10 and 12 years of age. This study helps establish that exergaming training has benefits similar to those of traditional exercise, providing a theoretical foundation for future research. However, to explore the relationship further, future studies should include a wider variety of participants and variables in order to yield more diverse results.

## Figures and Tables

**Table 1:** Demographic characteristics of the participants.

Variables	Total (*n* = 68)	EG (*n* = 35)	CG (*n* = 33)	*p*
Age (years)	10.76 ± 0.49	10.77 ± 0.49	10.76 ± 0.50	0.909
Gender (%)				0.994
Boys	48.5	48.6	48.5	
Girls	51.5	51.4	51.5	
Height (cm)	146.29 ± 9.40	146.70 ± 9.54	145.86 ± 9.38	0.715
Body weight (kg)	42.35 ± 12.91	40.65 ± 13.15	44.16 ± 12.58	0.265
BMI (kg/m^2^)	19.54 ± 4.74	18.54 ± 4.22	20.61 ± 5.09	0.071
BMI categories (%)				0.024 *
Obesity	22.73	8.82	37.50	
Overweight	6.06	8.82	3.13	
Normal weight	60.60	8.82	46.87	
Underweight	10.61	73.54	12.50	
Sleep quality (%)				0.446
Good sleep	55.9	51.4	60.6	
Poor sleep	44.1	48.6	39.4	

BMI = body mass index; CG = control group; EG = experimental group. * *p* < 0.05 is considered a significant difference between the experimental and control groups.

**Table 2:** Comparison of pre-test, post-test, and changes in sleep quality between the experimental and control groups.

Variables	Group	Pre-Test	Post-Test	Change	Group Effect	Time Effect	Interactive Effect
PSQI global score	EG	6.17 ± 2.96	4.80 ± 1.97 ‡	−1.37 ± 1.97 †	F = 0.147 *p* = 0.703	F = 11.884 *p* = 0.001 *	F = 5.903 *p* = 0.018 *
	CG	5.85 ± 2.37	5.58 ± 1.54	−0.27 ± 1.89			

CG = control group; EG = experimental group; PSQI = Pittsburgh Sleep Quality Index. † *p* < 0.05 is considered a significant difference between the experimental and control groups. ‡ *p* < 0.05 is considered a significant difference between the pre-test and post-test assessments within each group. * *p* < 0.05 is considered a significant difference in time and interactive effects.

**Table 3:** Comparison of sleep duration in pre-test and post-test assessments and changes between the experimental and control groups.

Variables	Group	Pre-Test	Post-Test	Change	Group Effect	Time Effect	Interactive Effect
Sleep duration (min)	EG	496.33 ± 68.01	505.15 ± 53.16 †	8.82 ± 61.09	F = 1.620 *p* = 0.207	F = 0.018 *p* = 0.895	F = 2.320 *p* = 0.133
	CG	489.94 ± 61.83	480.51 ± 54.13	−9.43 ± 45.62			

CG = control group; EG = experimental group. † *p* < 0.05 is considered a significant difference between the experimental and control groups.

## Data Availability

The data presented in this study are available from the corresponding author upon request.
